# Theory and Examples of Catch Bonds

**DOI:** 10.1021/acs.jpcb.4c00468

**Published:** 2024-04-18

**Authors:** Wolfgang Quapp, Josep Maria Bofill

**Affiliations:** † Mathematisches Institut, Universität Leipzig, PF 100920, Leipzig D-04009, Germany; ‡ Departament de Química Inorgànica i Orgànica, Secció de Química Orgànica, Universitat de Barcelona, Martí i Franquès 1, Barcelona 08028, Spain; § Institut de Química Teòrica i Computacional, (IQTCUB), Universitat de Barcelona, Martí i Franquès 1, Barcelona 08028, Spain

## Abstract

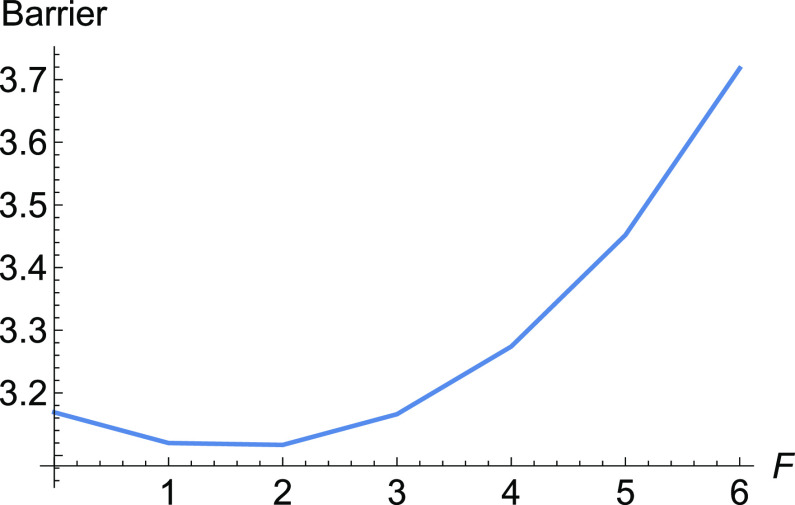

We discuss slip bonds, catch bonds, and the tug-of-war mechanism using mathematical arguments. The aim is to explain the theoretical tool of molecular potential energy surfaces (PESs). For this, we propose simple 2-dimensional surface models to demonstrate how a molecule under an external force behaves. Examples are selectins. Catch bonds, in particular, are explained in more detail, and they are contrasted to slip bonds. We can support special two-dimensional molecular PESs for E- and L-selectin which allow the catch bond property. We demonstrate that Newton trajectories (NT) are powerful tools to describe these phenomena. NTs form the theoretical background of mechanochemistry.

## 1 Introduction

Catch bonds were discovered 35 years ago,^1^ and they are observed in many biochemical molecules.^2,3^ As a molecule deforms, the rate of dissociation decreases. The stabilization of a complex by an external force in the catch bond model is an exciting phenomenon. To our knowledge, there are two proposals for suitable free-energy surfaces for modeling catch bond behavior.^4,5^ We will discuss these models as well as our own modifications. If one can assign a potential surface to the region of the molecule with presumed catch bond behavior, then one will be on a good theoretical basis for understanding the behavior.

We are convinced that the theory of Newton trajectories (NTs)^6,7^ is a tool to rationalize the biochemical phenomena of slip and catch bonds. To understand this mathematical theory, we assume that under an external force, the effective mechanochemical potential must be taken into account
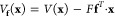
1where *V*(.) is the potential energy surface (PES) of a molecular system under consideration or its free-energy surface. **f** is the normalized direction of an external force vector acting on the molecule, and *F* is the magnitude of the force. The superscript *T* indicates the transpose. Note that force is a vector quantity, where both direction and size are important. By **x**, we depict the molecule in arbitrary coordinates. The approach of eq 1 is the simplest possible approach with a linear external force. Sometimes a nonlinear force of the form −*Fr* cos θ^8,9^ is used which we will not treat further here.

The stationary points of the PES move under the force. The new barrier of the effective PES changes with, but the barrier is a sum of two differences
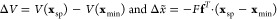
2The two parts can play together or can act against each other. This complicates the overall picture. The second part for Δ*x̃* in eq 2 has already been discussed in refs (2 and 10), for example. We mainly discuss the first summand below. Its solution curve is usually curvilinear. This contradicts the theory of Bell, as it is also discussed recently in ref (5).

The gradient of the effective PES for the stationary points, *V*_**f**_(**x**_*c*_), has to be zero to describe this movement of the stationary points, **x**_*c*_. It means that it has to apply
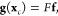
3where **g** is the gradient of the original PES. The value *F* changes like a parameter along the solution curve. Given a new value, say *F*′, a new point is found, **x**_*c*_^′^, in the manner for which eq 3 is satisfied for the new parameter value, *F*′. At each point **x**_*c*_, the parameter value, *F*, coincides with the root square of the gradient norm
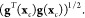
For the movement of any critical point, **x**_*c*_, one creates a differential equation^6,11^ by
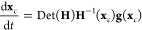
4where **H**^–1^ is the inverse of the Hessian matrix of the original PES, and Det(**H**) is the determinant of this Hessian matrix. A curve length variable, *t*, is also used. Solutions of eqs 3/4 are called NTs. Equation 4 was created a long time ago by Branin,^12^ see also the text book,^13^ where one uses the so-called adjunct Hessian. It is the desingularized matrix
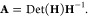
5The study by Barkan and Bruinsma^14^ also introduces parts of the theory of NTs; however, note that eq 4 is used in a probably misleading form in refs (5 and 14).

Each solution of eqs 3/4 to different directions **f** connects a minimum to a saddle point (SP) of index one, SP_1_. In chemistry, it is called a transition state (TS). More generally, an NT connects stationary points of an index difference of one.^15−17^ A solution curve has to cross a point on its path where it holds Det(**H**) = 0. The force in the direction **f** with the magnitude to reach the point with Det(**H**) = 0 forces the former minimum and former saddle SP_1_ to coincide. This event is called the bond breaking point (BBP).^7^ Each local point on the Det(**H**) = 0 manifold determines one solution curve of eq 3, though its corresponding gradient direction is there. The reason is that along every solution curve of eq 3, the gradient direction is fixed and is equal to **f**. The manifold Det(**H**) = 0 crosses anywhere on the PES, the manifold of valley-ridge inflection (VRI) points.^16,18−21^

The mathematical theory of NTs can be used to study several types of reaction paths, viz., the standard reaction model,^22^ as well as a reaction path under an external mechanical force^7,23^ and under an external electric field,^24,25^ the latter with a generalization of this theory. Some kinds of solutions of eq 3 can themselves serve as reaction pathway models, besides the well-known steepest descent model of the so-called intrinsic reaction coordinate,^26−29^ or the gradient extremals,^17,30−35^ or the gentlest ascent dynamics,^36,37^ or many other models.^38,39^

The aim of this paper is the foundation of molecular PESs for catch and slip bonds. For this, we propose simple 2-dimensional surface models. Special examples are selectins.^14^ The article is organized in the following way. In the next Section 2, we show the utility of the mathematical theory of NTs for the description of slip and catch bonds under an external force. Section 3 describes the tug-of-war mechanism based on the NT theory. We numerate different models with capital letters, A–F. After a discussion in Section 4, there is reported in Section 5 a glossary of definitions of biomechano-chemistry. Finally, in Section 6, a set of conclusions is reported based on the present models. Formulas and tables are added in an Appendix.

## 2 Slip and Catch Bonds

### 2.1 Slip Bonds

There are different types of bonds: one type is the ideal bond^40,41^ being insensitive against tensile forces. It has been suggested to play a role in enabling the receptor–ligand pair to withstand tensile force, but it has not yet been reported in experiments.

Usually bonds weaken under the action of a mechanical load. Such chemical bonds, whose dissociation rate grows with an increasing external force, are called slip bonds. The name was coined by Bell in 1978 for biological adhesive bonds.^42^ An example is B cells which apply forces to segregate and rupture clusters and individual antibody–antigen interactions which exhibit a slip bond character.^43^ Thus they show lifetime reduction under force. Unbinding forces of single antibody–antigen complexes correlate with their thermal dissociation rates. The same TS must be crossed in spontaneous and forced unbinding. The unbinding path under load cannot be too different from that at zero force.

**Figure 1:**
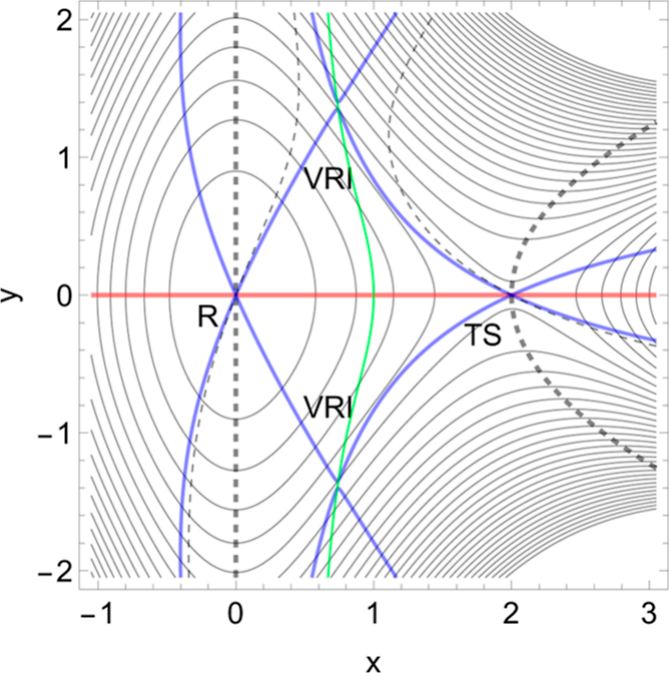
2D PES (of eq 8 in Appendix) in the forceless limit, *F* = 0, depicted by thin equipotential lines. *R* is at the zero level, but the SP is at 4 energy units. The red curve is a typical slip bond NT, the thick dashed one is a putative catch bond NT, and the thin dashed one is no direct catch bond NT (see point B in subsection 2.2 below). The blue curves are singular NTs through the two VRI points. The green curve is the Det(**H**) = 0 line of the PES which also crosses the VRI points.

Let us assume the PES landscape, *V*(**x**), of a molecular system comprised of a receptor and a ligand linked by a noncovalent bond. Coordinates (*x*, *y*) may be interesting distances or angles of the molecule. The bound state corresponds to a minimum of the PES named the reactant, *R*, in Figure 1. [All drawings and calculations are done with Mathematica 13.3.1.0 for platform Linux x86 (64-bit).]

The application of a tensile mechanical force, *F***f**, in the direction (1, 0) of the red NT moves the minimum, *R*, and the TS together. It decreases the height of the barrier if *F* increases from zero to some value, implying an increase of the dissociation rate. As a result, the lifetime of the slip bond decreases when it is stressed by a tensile force.^44,45^ When the external force is applied, then the effective PES, *V*_**f**_(**x**), describes the evolution of the bound state to the unbound state, and it is given by eq 1.

Slip bonds depict directions where the corresponding NT to **f** leads more or less directly from a minimum to a TS. A typical case of a slip bond is shown in Figure 1 by the red NT. It leads from the reactant *R* over the SP to the dissociation. Its direction is (1, 0). At a final amount of *F*, the former minimum and the former SP_1_ coalesce to a shoulder point, which is the BBP on the original PES. There the green curve crosses the red NT. In the original PES, the positive curvature of the minimum bowl turns over into the negative curvature at the SP.

### 2.2 Catch Bonds

Seen from the TS, the slip direction is the natural direction of the SP valley; see the TS on the red *y* = 0 line in Figure 1. Of course, one can deviate to both sides up to a certain degree, and one still goes downhill in the reaction valley. It would still hold the slip bond character. However, if the external excitation direction becomes more or less orthogonal to the col, then one goes uphill. Such a case was then named the catch bond direction if the barrier increases under the external force.

Note that for an *N*-dimensional PES, we have a one-dimensional valley path over the SP_1_, the slip direction; however, we have (*N* – 1) directions orthogonally uphill into the PES mountains.

An early work^1^ describes the occurrence of states in which adhesion cannot be reversed by application of tension. Such states occur only if the adhesion molecules have certain constitutive properties, thus having a catch bond character. Often catch bond behavior is connected to sheer forces in proteins.^46^ Another example is dynein’s interaction with microtubules which behaves like a catch bond.^47^ The dependence on the force direction to modulate the multistep process of translation is reported.^48,49^ The calculated forces alter the TS barrier of the peptidyl transfer reaction catalyzed by the ribosome for two alanine residues in the peptidyl transfer center as a function of the direction of the force applied to the P-site residue. In a current discussion,^50^ vinculin is a load-bearing linker protein that exhibits directional catch bonding due to interactions between the tail domain and the filamentous F-actin, see also refs (51 and 52). There is a large amount of reports on catch bonds.^2,3,53−63^

(A) Molecular examples: selectins

**Figure 2:**
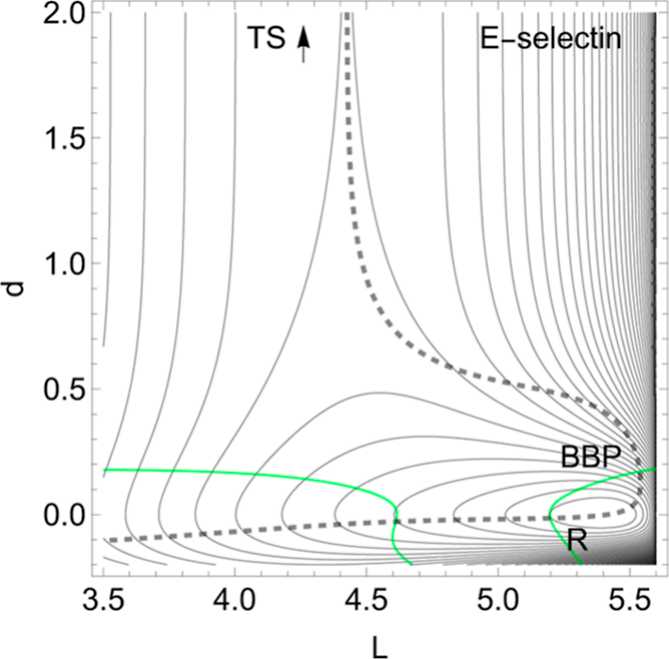
Proposed 2D PES of E-selectin with eq 6 in the Appendix, in the forceless limit, *F* = 0, depicted by thin equipotential lines by steps of 7.4 pN. *R* is the global minimum. The TS of the dissociation limit is far outside the scale. The thickly dashed NT to direction (1, 1) is a slip-catch-slip bond NT given in ref (5). The green curves are the Det(**H**) = 0 lines of the PES.

First we report a current example of catch bond behavior in endothelial selectin (E-selectin). Let us assume the simplified 2-dimensional landscape, *V*(*L*, *d*), of a molecular system^5^ constituted for the receptor of E-selectin and the ligand sLe^*x*^ linked by a noncovalent bond.^64^ It is a challenge to select special coordinates when dealing with protein–ligand interactions. We find high dimensionality and great flexibility. Here^5^ one uses two length coordinates, *L*, *d* in nm, to describe the movement of the ligand. *L* is the protein extension, but *d* is the distance to the ligand. Forces are given in pN. The bound state corresponds to a minimum of the PES named reactant, *R*, in Figure 2. The TS is the final level of a Morse ascent at large *d* and *L* ≈ 4.44 nm.

**Figure 3:**
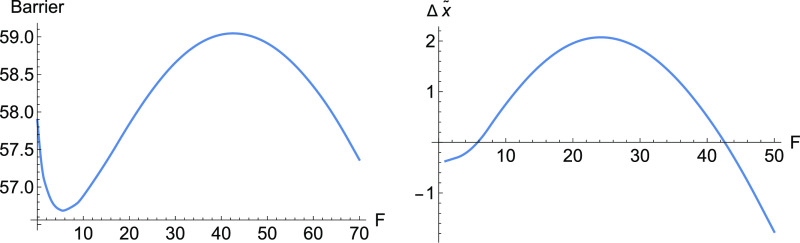
Barriers for E-selectin for increasing force, *F*, along the NT to direction (1, 1). To the left is the full barrier, but at the right panel is, for comparison, shown the influence of Δ*x̃*.

In both the molecular examples, in this point (A), we mainly treat the direction (1, 1) for the external force; note that we do not normalize this vector by the , for simplicity. The application of eqs 3 and 4 for a tensile mechanical force, *F*(1, 1), along the dashed NT, decreases the height of the barrier if *F* increases from zero to 5 pN. Then the barrier begins to increase, up to 40 pN. After this value, the barrier again decreases, like in a slip bond, see Table 1 in the Appendix and the right panel of Figure 3. The barrier is a nice, typical characteristic of catch bond behavior. It finally needs here a very large force up to ≈350 pN for a full bond breaking where the former TS and the former minimum, *R*, coalesce at the BBP, the crossing of the NT and the right Det(**H**) = 0 line (the green line).

When the external force is applied, then the effective PES, *V*_**f**_(*L*, *a*), describes the evolution of the bound state to the unbound state and is given by eq 1. Here, at the early beginning of the external force, *F*(1, 1), the Morse TS becomes lower and the minimum, *R*, becomes higher. It means Δ*V* in eq 2 decreases, and we have a slip behavior. Later, probably the catch bond character is coming from the second part of eq 2, and compare both barriers in Figure 3. Because there is only the Morse TS of the dissociation, in this example, the variation of the coordinate *d* is large for moving stationary points, under force *F* along the direction (1, 1). This causes a large part of the Δ*x̃* in eq 2. After *F* = 40 pN, the Δ*x̃* becomes negative, and we find again slip behavior.

An **indicator** for a catch bond can be a large movement of the involved stationary states of the PES under external force.

Note that the dashed NT has a turning point (TP) at (*L*, *d*) ≈ (5.4, 0.4). This must not be directly connected to the catch bond character, see also an extreme counter example in ref (6). However, the existence of a TP is also an indicator of a possible catch bond behavior. An NT between *R* and TS without a TP describes a slip bond, like the red NT in Figure 1.

**Figure 4:**
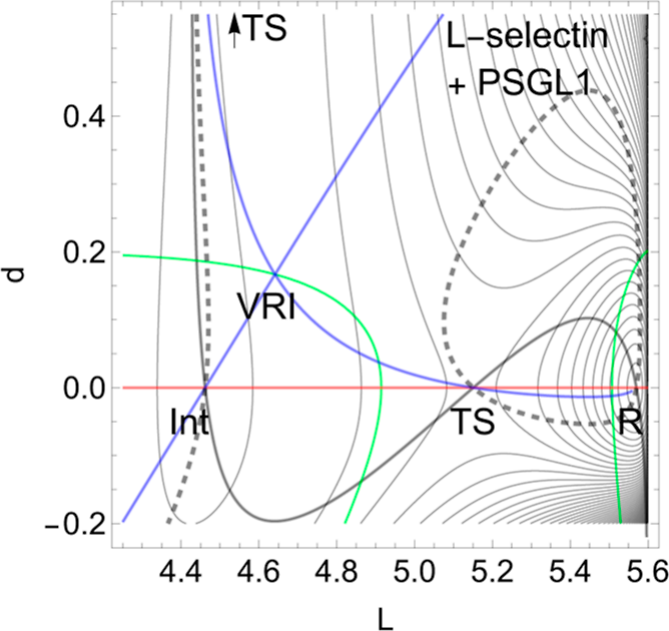
Proposed PES of L-selectin^5^ with eq 7 in the Appendix. The TS of the dissociation limit is far outside the panel. The thickly dashed NT to direction (1, 1) is the NT with catch bond behavior. The red line is the NT to direction (−1, 0), and the gray-black NT goes to direction (−1, 1). Both are slip directions. The blue NT goes through the VRI point; it is the separatrix for the catch bond NT. The green curves are the Det(**H**) = 0 lines of the PES.

A second nice example is leucocyte selectin (L-selectin); see Figure 4. Now the situation on the PES dramatically changes because a new TS emerges on the *L*-axis at (5.18, 0). The model concerns the parts of eq 2 in a more mixed form, but Δ*x̃* again plays the main role. For the chemical details, see refs (3, 5, 65 and 66), as well as for the representation of the molecule. The formula of the 2D model PES is given in the Appendix in eq 7. Note an analog formula like in the E-selectin, only the parameters are quite different. There two length coordinates, *L*, *d* in nm, are used to describe the movement of a ligand. *L* is the protein extension, but *d* is the distance to the ligand. Forces are given in pN.

On the *L*-axis, we have two minima and one TS. This axis is also an NT to direction (−1, 0) drawn in red. It is a slip direction. The right minimum, *R* at (5.58, 0), is the global minimum, but the left one at (4.48, 0) is a flat intermediate. Along increasing *d* on the line with *L* ≈ 4.43, we have a dissociation channel of the ligand. Thin lines of the figure are level lines with steps of 5.2 pN. The green lines are the Det(**H**) = 0 lines of the PES.

**Figure 5:**
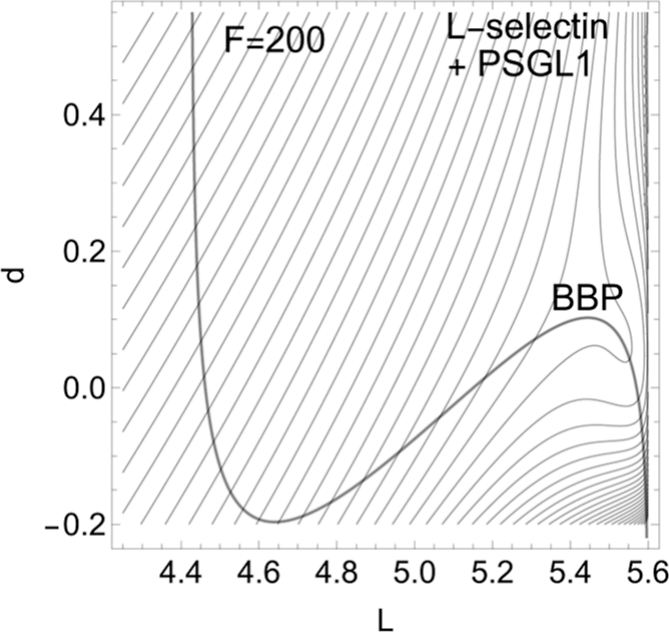
Effective PES of L-selectin for the gray NT to direction (−1, 1) of Figure 4, for force *F* = 200 pN. The former TS and the former minimum, *R*, now form a shoulder. The barrier disappears.

Of interest is the gray-black NT to direction (−1, 1) from *R* over *TS* through *Int* to dissociation. It should be the usual direction of the process of interest: globally, *L* decreases, but *d* increases along (−1, 1), if the ligand moves away. It has slip bond character, because along this NT, under increasing force, *F*, the barrier decreases. Along the NT, under force, the minimum, *R*, and the TS move toward each other, and this causes a decrease of the barrier. If one exits the system up to *F* = 200 pN, then minimum *R* and TS coalesce at a barrier breaking point (BBP), see Figure 5. It is the crossing of the NT and the green line in Figure 4.

On the PES of Figure 4, through a VRI point at (4.65, 0.17) is calculated a singular NT which is drawn in blue. The VRI point marks the border between the two (orthogonal) valleys to *R*, or to the dissociation TS, and the singular NT separates the two thickly dashed branches of the NT of special interest here. It goes to an orthogonal direction, (1, 1), with a pulling of the molecule along both coordinates. The NT direction is shown by black dashes. The pulling along *L* seems to be counterproductive for the process. Seen from *R*, the pulling points into a dead valley on the right side of the figure. The right closed branch of the dashed NT connects only *R* and the *TS*. Its upper arc is disrupted from the left branch through *Int* by the blue, the singular NT. This NT acts as a separatrix.^5^

It is a second **indicator** of catch bond behavior: The main exit to the dissociation and the NT of interest through the minimum, *R*, are divided by a singular NT.

If one excites the molecule along direction (1, 1), then one moves the stationary points *R* and *TS* along the upper arc of the dashed NT. Here we find catch bond behavior, see Table 2 in the Appendix. Why? The minimum bowl is deep and thus has strong curvatures. Its eigenvalues in *L*- and *d*-directions are ≈(15,300, 4850) but the eigenvalues at the *TS* are ≈(−280, 570). Thus, any movement along the given excitation for *F* up to ≈60 pN will move more the TS rather than the minimum. That is why the second part of eq 2 does play the main part here. The change in Δ*V* is only 1/5 of the change in the barrier. For higher *F* values, however, the arc over *TS* and *R* closes, and we go back to slip behavior to a decreasing height of the barrier. And at the end, if *F* is so large that the BBP on the green line is reached from both sides, then the molecule finally slides to dissociation, see Figure 5. We guess that the 2D example of Figure 4 will be the main pattern for catch bonds. It is already anticipated by a schematic picture by Figure 2B in ref (3).

Of course, by drawing a comparison of the catch bond NT, the thickly dashed upper arc to direction (1, 1), its slip bond counterpart below to direction (−1, −1), and another usual slip bond NT to orthogonal direction (−1, 1), the gray-black one, this shows that one has an “asymmetry” for the catch bond.^67^ This requires no further reasoning. One cannot expect that the catch bond property holds for any direction of the external force. Thus, it does not exist as “the catch bond”, but a bond can have “catch bond character”.

In the following, we discuss further different scenarios for the force-induced change of the TS of a simple two-dimensional (2D) PES, especially for an inversion of the relations between the curvatures in the minimum and TS, in comparison to the former example. This approach was proposed by Suzuki and Dudko.^4^

(B) Linear reaction valley

A very simple abstract model PES with one minimum and one TS on a linear reaction channel is obtained by eq 8 given in the Appendix; see Figure 1. On the right-hand side, we have a dissociation exit. We have in Figure 1 the level lines (thin black) and some NTs. The red one is a typical case of a slip bond depicted by an NT to the *x*-direction. The application of a tensile mechanical force, *F***f**, in the direction of the red NT to the direction (1, 0) moves the minimum, *R*, and the TS together. It decreases the height of the barrier if *F* increases from zero to some value, implying an increase of the dissociation rate. At the same time, it also decreases the coordinate *x* of the TS. As a result, the lifetime of the slip bond decreases when it is stressed by a tensile force.^44,45^

**Figure 6:**
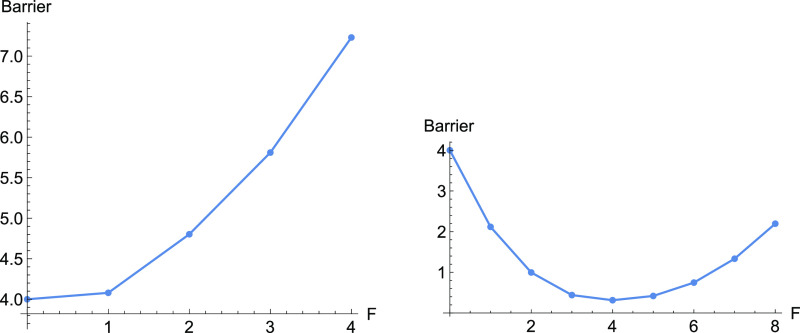
Barriers along the two dashed NTs of Figure 1 with increasing force, *F*. To the left is the thick NT, but at the right panel the thin one.

If one uses an excitation in pure *y*-direction, (0, 1), then one obtains two branches of the bold dashed NT. Some values for the force *F* in this direction are given in Table 3. A typical increase of the barrier with force takes place in the left panel of Figure 6.

A **third indicator** for an increase of the barrier is here that the curvature at the TS across the reaction channel is larger than the curvature in the reactant in the same direction.

The eigenvalue in the *y*-direction in the minimum, *R*, is 2 but in the TS, it is 10.

Again, the condition is that the NT is indeed more or less orthogonal to the reaction valley to go outside the blue separatrix. It is secured here because additionally the dashed NT is ruptured into two branches; one goes through the minimum, but the other one goes through the TS. The border cases are the singular NTs (blue curves) through the VRI points. They depict excitation directions (±1.315, 1), and they form the separatrix. There are two such NTs in our example. Inside the region of these singular NTs between the VRIs are only directions for excitations with slip bond character, like the trivial red NT. The situation changes for the other side of the singular NTs for the dashed NTs. They are disrupted by the VRI points.

However, if the excitation direction of an NT is too near to the valley direction, though the NT is above the VRI points, then also a slip behavior can be observed, at least for low *F* values, see Figure 6, at the right-hand side. The used thinly dashed NT depicts an excitation direction (0.75, 0.64). Additionally, here acts the shortening of *x* for the thin dashed NT, and the extension of *x* for the thick dashed NT, if we leave the TS. This is again the action of the right part of eq 2.

One important observation, however, concerns both dashed NTs: they are divided into two branches and go uphill to infinity on the abstract PES. Therefore, the moving minimum and the moving TS can never coalesce under the excitation force. It means that they can never end in the usual finale of a slip bond in which the barrier disappears. Belyaev and Fedotova^3^ name this an ideal catch bond, in contrast to the realistic catch–slip bond of real molecules.

We have to notice that although the dashed NTs show (from the beginning or later) an increase in barrier height, they do not show the true character of the realistic catch–slip bond. The possible increase of the barrier in a one-valley model was already reported by Suzuki and Dudko in 2010.^4^ (We did not understand this in our 2016 paper^6^ where we treated only a slip NT.) However, that the possible catch bond NTs in this one-valley model will not come back to a final decrease of the barrier, means a disturbing insight. It later leads us to the next section, to the two-valley model.

(C) Curvilinear reaction valley

**Figure 7:**
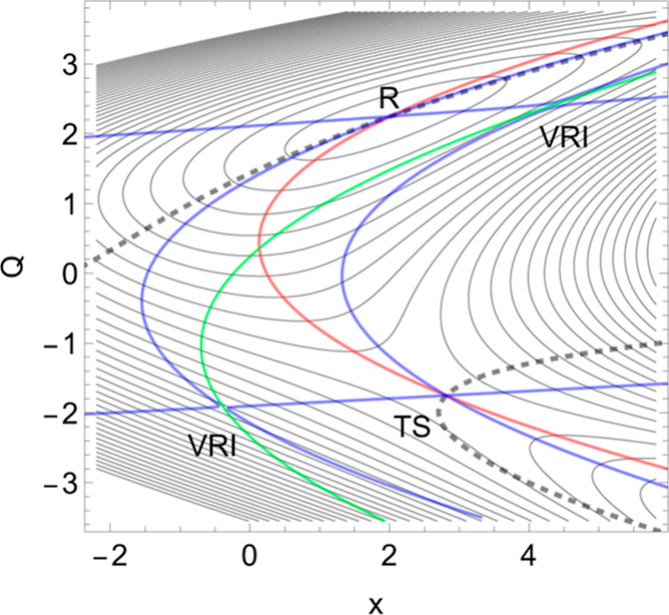
2D PES of eq 9 in the forceless limit, *F* = 0, depicted by thin equipotential lines. *R* is at −60 energy units, but the SP is at +60 energy units. The red curve is a typical slip bond NT, but the two blue curves are singular NTs through the two VRI points. The dashed curve is an NT divided into two branches which are of interest for a catch bond behavior. The green curve is the Det(**H**) = 0 line of the PES.

This example, Figure 7, is quite similar to the linear case above. We treat a nonlinear case of the dissociation valley, with a PES model proposed by Suzuki and Dudko,^4^ see eq 9 in the Appendix. Coordinates (*x*, *Q*) are again the interesting distances or angles of the molecule. (We have adapted some parameters of the model.) The reactant *R* is separated from the unbound state by an SP_1_, an SP of index one, located on top of the PES barrier depicted by the TS. The long flat minimum valley ends at both sides by dead valleys, but the dissociation exit comes over a side valley, see Figure 7. The situation is similar to the global minimum of the Müller–Brown surface.^68,69^

Shown in Figure 7 are also two (blue) singular NTs that cross two VRI points. They are again the border lines for NTs with slip bond character. All the directions between the two singular NTs are allowed for slip bonds, where the more central NTs, which are like a steepest descent from the TS, are of course the better ones. Such “good” slip NTs do not have a TP on their energy profile;^7,14^ however, a TP does not disturb the slip character in the region between the VRI points, see an extreme case in ref (6). This remark contradicts the treatment by Barkan and Bruinsma^14^ where the border for slip bonds is any *f*-switch point.

**Figure 8:**
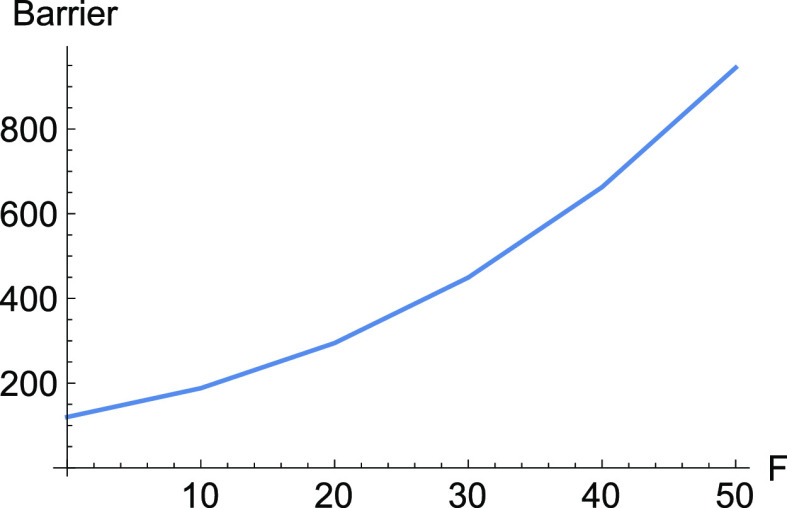
Increase of the barrier for the dashed NT of Figure 7 with increasing force, *F*.

In Figure 7, a candidate for a catch bond NT is shown by the black, dashed NT. Its direction is **f** = (0.55, 0.83). It is divided by singular NTs into two branches. One branch leads from *R* along the minimum valley in the upper parts of the figure, but the other part crosses the TS and connects the right ridge with the exit valley to the lower right corner. These branches of this NT describe the movement of the two stationary points, minimum and TS, on the corresponding effective PES if the external force *F***f** is applied. One branch describes the movement of the minimum, but the second branch describes the movement of the TS. The external force now increases the difference of the energy between the minimum and SP_1_. The reaction rate will decrease. In this example, the effect is dramatic; see Figure 8 and Table 4 in the Appendix.

**Figure 9:**
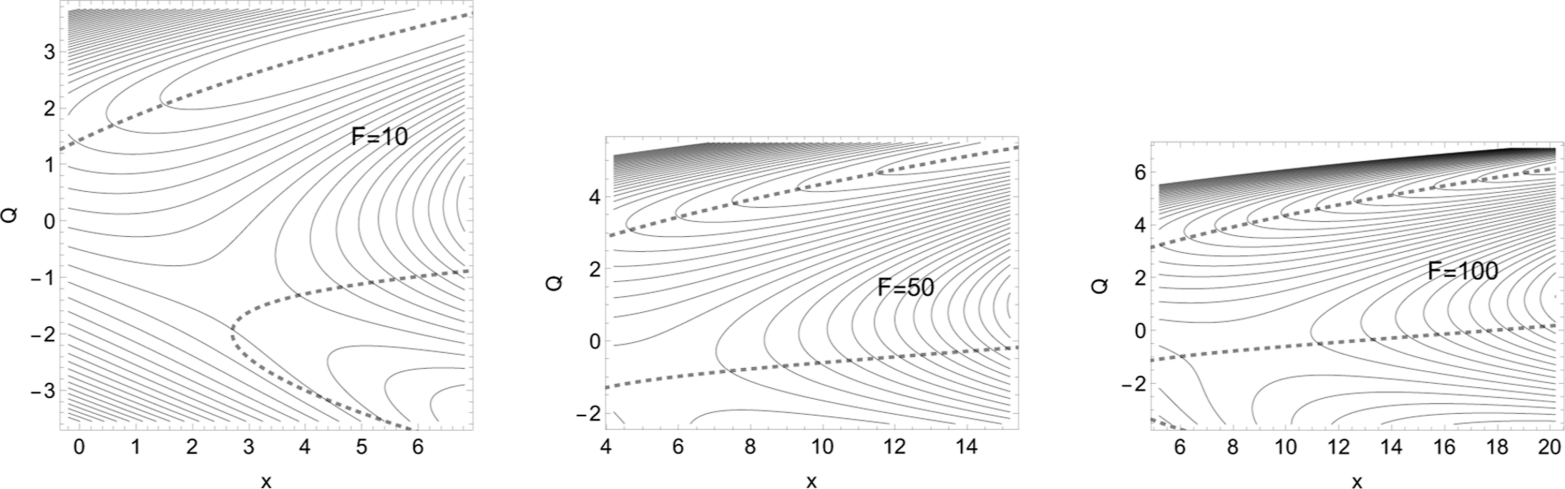
Three cases of an effective PES to eq 9 with external force along eq 1. The direction of the force is that to the dashed NT of Figure 7, and the amounts are *F* = 10, 50, and 100 force units. Note the movement of the sections shown along the *x* axis. The stationary points move on the corresponding NT; the minima in the last two cases are far out off the figure, see Table 4.

Although we have a dramatic increase of the barrier height of the example of Figures 7 and 8, we cannot say that it shows true realistic catch bond character because it does not turn back after a certain amount of force, *F*. The two branches of the dashed NT never cross. They cannot allow the minimum and the TS to coalesce. Different cases of the effective PES are shown in Figure 9.

(D) Curvilinear reaction valley with an emerging shoulder

**Figure 10:**
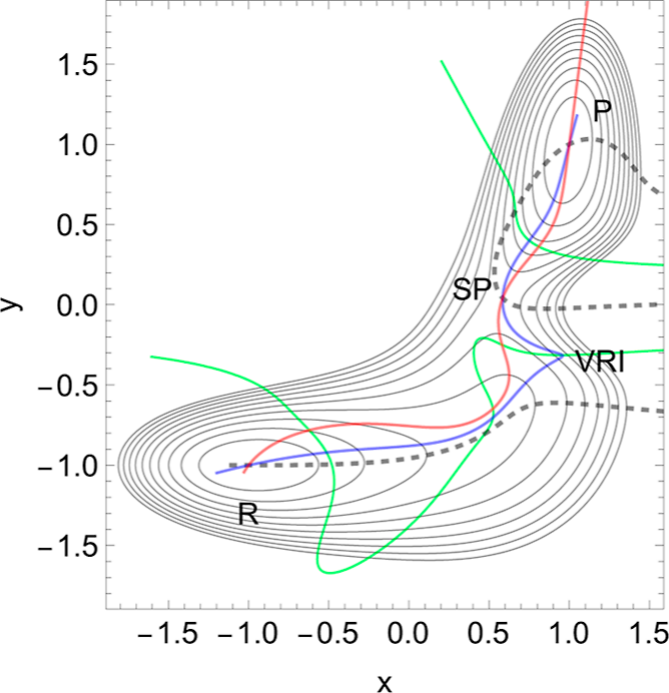
PES to eq 10 with two minima and a TS in between. A disrupted black dashed NT for the catch direction (1, 0) is shown. Further NTs are with directions red (0.15, 0.99) for a slip bond character and blue (0.67, 0.74) for the singular NT. The TP of the singular NT is the VRI point of the PES. It again divides the two branches of the dashed NT.

We use a PES, Figure 10, along with eq 10. Now we find a reactant, *R*, an SP, and a product minimum, P. The key for our treatment is again the narrowness of the col. The red curve is again an NT leading from the reactant, *R*, over the SP to the product, P. Its direction is (0.151, 0.989). The blue NT is a singular NT through the VRI point, with the direction (0.646, 0.711), and the black dashed NT is a putative catch bond NT. The force for this NT is assumed in the *x*-direction. The green curve is the line of points with condition Det(**H**) = 0 for the Hessian matrix. It crosses the reaction valley from the minimum to the TS three times. This property will lead to an interesting behavior of the PES under external force in the *x*-direction: in the long, gently rising valley of the reactant, *R*, emerges under *F* = 6 units of force a further minimum. It replaces the former shoulder, before the TS. So to speak, a population of states of the former global minimum splits into a bimodal population for some time, as it is reported in ref (70).

At the beginning of such a force, we get a slip bond behavior between the reactant and the TS. But at *F* = 12 units, the new minimum becomes the global one, and its relation to the TS now turns to catch bond behavior. The corresponding stationary points of the effective surfaces are given in Table 5.

**Figure 11:**
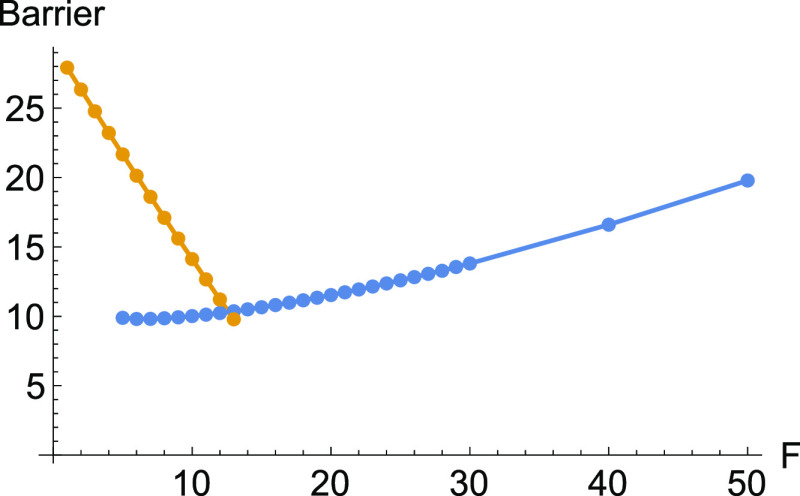
Two curves for the barrier height of the effective PES to eq 10 with an external force along the *x*-direction. Dark yellow is the barrier to the original global minimum, and the blue curve depicts the barrier to the new minimum.

In Figure 11, we report the barrier height between the two minima and the TS under different forces, *F*. The dark yellow line concerns the original minimum, but the blue curve of points shows the difference from the new one.

**Figure 12:**
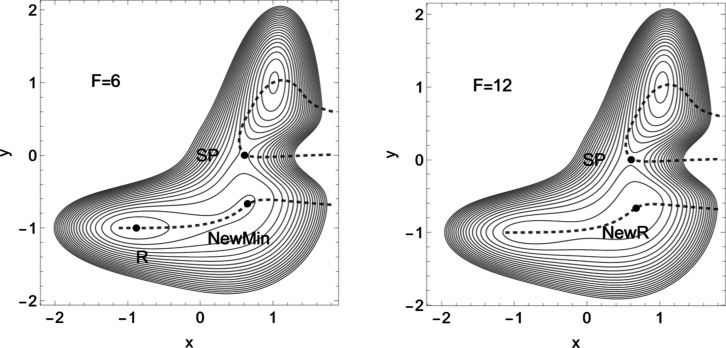
Two cases of an effective PES to eq 10 with external force along eq 1 in *x*-direction, the dashed NT of Figure 10, and the amounts are *F* = 6 and 12 units. The stationary points move on the corresponding dashed NT.

In Figure 12, we report the two effective PESs under different forces, *F*. The appearance of the new minimum is to detect.

An indicator for the catch bond character was that the corresponding curvatures in the NT direction are both positive, but the one at the TS should be larger than that at the minimum. Here we have the eigenvalues of the Hessian at the minimum of (80, 402) and at the TS of (307, −157). In a contrary case, the difference of both energies will decrease and the catch bond character does not appear.

In all three cases B–D of different mathematical test PESs, we obtain a desirable increase of the barrier under a corresponding external force. However, it happens along disrupted NTs (the dashed ones), which go up to infinity on the simple PES, so that a final decrease of the barrier can never take place. These NTs cannot be correct models for the observed behavior in mechano-bio-chemistry, namely, the final catch-to-slip behavior of the reported experiments.^71^

Summary of subsection 2.2

In this subsection, we studied different models:

The cases (A) of E-selectin in Figures 2 and 3, and L-selectin, Figures 4 and 5, allow a catch–slip bond property. The next cases B–D show the catch bond behavior; however, they do not come back finally to the chemical slip behavior. So, they can be seen for abstract models, where for real molecules, the border properties have to still be changed.

### 2.3 Pure One-Dimensional Path Model

There is another one-dimensional path model to explain catch bonds.^72^ However, we assume inappropriate use of a rate formula. One uses Kramers’ rate. This approach does not apply to a disappearing barrier.^73^ The error also concerns the rate formula eq 41 of ref (74). The inadequacy of the formula used was already reported in 2006,^75^ but it is currently in use again.^76,77^

From the beginning of the treatment of the catch bond problem, a 2-pathway model was proposed for it.^54,67,78−85^ We propose to accept such an explanation. The catch bonding appears to be an important phenomenon that can occur when multiple valleys are present. Slip bonds then work together collectively;^62,86^ see below the situation in the tug-of-war mechanism where we use the case of two reaction valleys.

## 3 Tug-of-War Mechanism with a Catch Bond

The jump from one given reaction valley to another is a central task for the application of external forces. There is a long list of experimental results concerning this tug-of-war mechanism^47,70,87−90^ to name a few. It also concerns the stereoselectivity of reactions.^71,91^ Further examples of the tug-of-war mechanism are unifying mechanosensing and affinity discrimination. T cells employ multiple modes of mechanical proofreading to stabilize receptor binding and achieve specificity of discriminations. These include catch bond behavior during activation^92^ and negative selection^93^ or conformation changes of adhesion molecules.^94^ In contrast, B cells apply forces which exhibit a slip bond character.^43^ Catch bonds drive stator mechanosensitivity in the bacterial flagellar motor.^95^ For another molecular motor model, a “tug-of-war” solution was proposed in ref (96). Molecular motor proteins use the energy released from ATP hydrolysis to generate force and transport cargo along cytoskeletal filaments. This suggests that dynein’s interaction with microtubules behaves like a catch bond.^47^ We note that the expression “tug-of-war” may be coming from the molecular motor problem for dyneins.

If there are two competing TSs for two possible reactions from one reactant, then a VRI point^6,7,16,18,19,22,97−100^ is always in between; compare in detail in Figures 2 to 9 of ref (7). The VRI point divides the two valleys of the PES.^21,101−105^ It is characterized by a special kind of NT, the so-called singular NT that bifurcates at the VRI point. The bifurcation then has four branches. From the minimum, one has a branch to the VRI point, from there, two branches to the two next SP_1_, and one branch usually goes further uphill to an SP_2_, an SP of index two. For the 2D toy surface, it is usually a maximum. Again, families of regular NTs connect an SP_1_ with an SP_2_, stationary points with an index difference of one. Seen from the minimum, a slip direction for one among both the TSs of index one on the PES cannot be the slip direction for the other one because no two NTs of the two families can start from the minimum with the same direction. To every family belongs a separate region of directions. In a minimum of a 2D surface, all “360*°*-directions” are possible; however, the full range is divided by special singular NTs through the corresponding VRI points.

(E) A simple example

**Figure 13:**
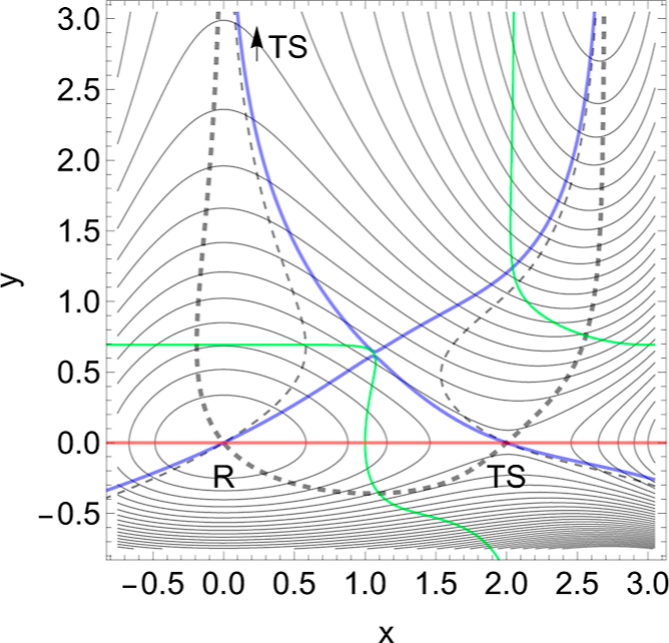
PES similar to Figure 1 with NTs, dashed, red and blue as above. The difference is a Morse potential along the *y*-axis.

Figure 13 demonstrates the tug-of-war mechanism. The PES is described by eq 11 in the Appendix. It is similar to Figure 1 but here, the *y* direction is replaced by a Morse potential. The PES is a simple model because the directions to different SPs are orthogonal from the beginning, like in ref (90). Left below may be the reactant structure, *R*. The second dissociation channel goes along the *y* axis. On the right-hand side emerges a ridge. The curvatures at the minimum, *R*, are represented by the eigenvalues (6, 20) but they are (−6, 27) at the TS, thus they are again different, and across the TS, the curvature is larger than across the minimum.

If one selects the direction (−0.24, 0.97) for an excitation and one goes uphill from *y* = 0, then the bold dashed NT again describes the pathway of the moving stationary points, *R*, TS1, and new TS2, the Morse finale level at (0, ∞). Coordinates, energies, and barriers for different forces, *F*, are given in Table 6. This direction still will be the catch direction for the former SP at (2, 0); however, it will be a slip direction for the dissociation for the Morse direction along the *y* axis. Quickly, near force *F* = 2, the former Morse exit level becomes lower than the former TS on the *x* axis, thus the *y*-dissociation becomes the reaction coordinate.

The red NT in Figure 13 along the *x*-axis describes the typical slip bond direction; however, even the inverse direction of the bold dashed NT becomes a slip direction.

(F) PES with 4 minima: Slip-catch-slip case

In early papers on selectin catch-slip kinetics,^106,107^ a slip-catch-slip behavior has been reported. It concerns protein E-selectin. The corresponding proposed PES of ref (5) shows only one valley from bound state to dissociation, as in the example of Figure 2. The slip-catch-slip behavior on this PES may be caused by the Morse TS that is far away from the reactant, and the movement of this TS under force may cause large changes in Δ*x̃*.

**Figure 14:**
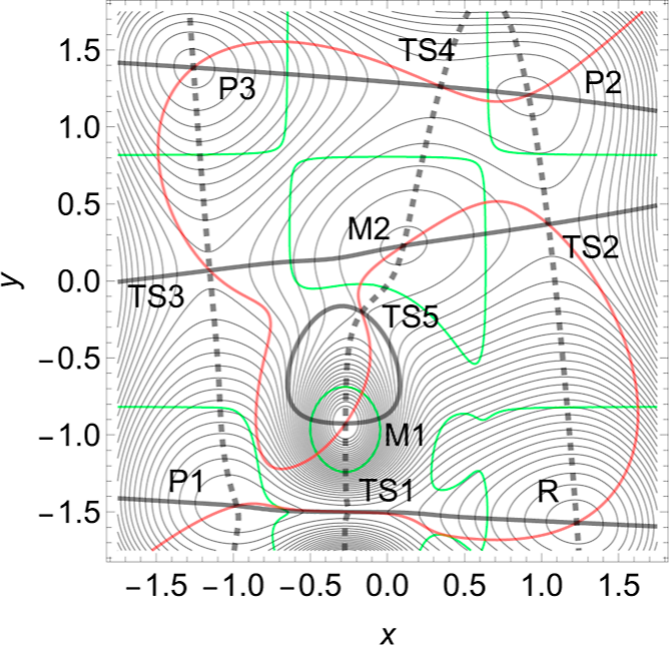
Modified Wolfe-Quapp PES, see eq 12. Three NTs are given, see text. Two maxima arise here, M1 and M2. Note that the curvatures in *y*-direction are different in *R* and TS1 by a factor of ≈10.

We propose an abstract 2D model PES to enforce slip-catch-slip behavior for smaller changes in the coordinates. For a better understanding, we first notice that the indicator of a strong curvature difference between the reactant and catch bond TS is not sufficient. To demonstrate this, we have chosen a modified Wolfe-Quapp surface, see Figure 14. The formula in eq 12 is given in the Appendix. Between the former maximum, M2, of the Wolfe-Quapp PES, and the TS1, an additional strong hill M1 is included. A further TS5 emerges between the hills.

There are shown in Figure 14. The dashed one points in the *y*-direction, (0.075, 0.997) and the black one in the *x*-direction (1, 0), but the red NT between, in the direction (0.74, 0.67). The red NT is used for comparison for certain skew NT directions. It could provide a slip direction for both reaction paths starting in *R*. As above, the green lines are the curves of BBPs. The solid black NT would depict an excitation into the *x*-direction, where this is actually the usual reaction valley from the reactant minimum, *R*, over TS1 to P1. However, we will excite the system into the *y*-direction and thereby force the reaction via TS2.

**Figure 15:**
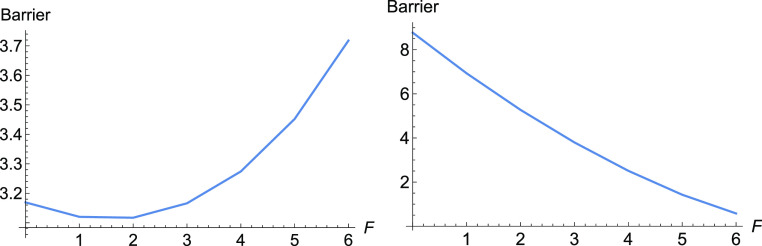
Effective barriers between *R* and TS1 and *R* and TS2 (right) for the *y*-excitation direction, on the PES with 4 minima of Figure 14. The two barriers swap their order. The curves cross, and later TS2 disappears.

The eigenvalues of the Hessian at stationary points of interest point along the axes, and they are at the reactant minimum, *R*, (12.99, 21.71), and at the TS1, they are (−4.24, 218.02). So, in the *y*-direction they are quite different, and the one at the TS is much larger. The curvature is chosen to be larger by a factor of ≈10. Nevertheless, at the beginning, the excitation to the dashed direction works at the TS1 like a slip bond. The barrier between *R* and TS1 decreases up to a force of *F* = 2, see Table 7 and the left panel of Figure 15. The reason is the action of the second part of eq 2. However, the strong hill of the maximum, M1, enforces at least a catch bond character for the barrier of TS1, for further growth of *F*, see Table 7. Because with increasing *F* beginning from *F* = 3, the barrier increases, compare Figure 15.

**Figure 16:**
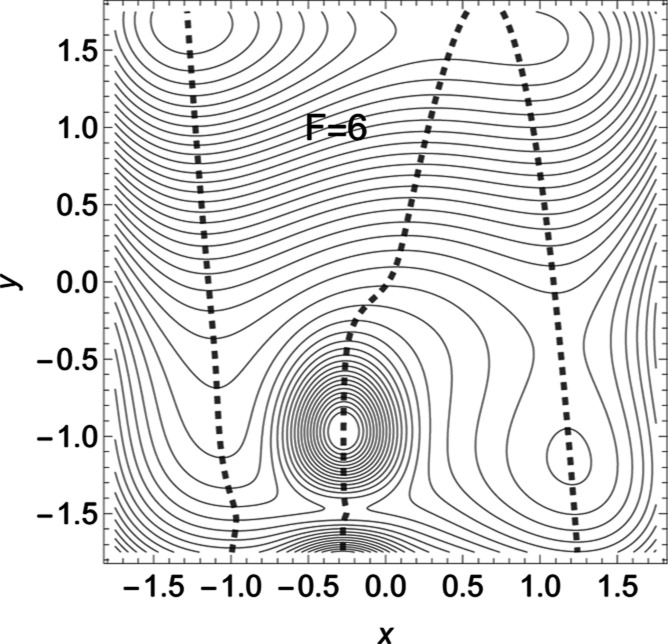
Modified Wolfe-Quapp effective PES for *F* = 6. The dashed NT describes the movement of the stationary points in direction **f** = (0.075, 0.997). The TS2 slowly disappears.

Later, for *F* = 7, the direction to TS2 opens for a main reaction and the barrier TS2 disappears. We still give the effective PES in the case *F* = 6 in Figure 16.

## 4 Discussion

There are some interesting conceptual models for catch bonds in Figure 2 of ref (2), see also Figure 7 of ref 108^108^ and Figure 1 of ref (9). We should note that the connection to our abstract mathematical models of PESs is still open in the general case. A next treatment should try to find appropriate coordinates for the conceptual models of ref (2) as it is done in a recent paper^5^ for L-selectin. However, this work^5^ has errors in its mathematical execution. It will be discussed in a following paper.

Another problem remains, i.e., how our theoretical findings could be translated into the practical mechanochemical process, for example, by hydrodynamic forces^109^ or in a ball mill.^110,111^ In which direction does a ball mill or grinding work? They are intrinsically isotropic techniques.

The addition of further outer excitations becomes even more complicated: thermo-mechanochemistry, sono-mechanochemistry, electro-mechanochemistry, and photomechanochemistry.^112^ A possible clue could be the principle of Le Chatelier: Any vibrational excitation of the molecule by a random external force, like ball milling, may probably excite the energetically lowest normal modes first.^113^ One could therefore tend to the result of ref (114) that the reaction mechanism for the process under ball-milling conditions is the same as in solution.

In the case of the PES of Figure 10, this could be the catch bond in the *x*-direction. In the case of the surface of Figure 13, this would be the usual reaction direction because the TS1 is lower than the TS2. Here we assume that ball milling would not have an extra effect.

## 5 Glossary: Mechano-Bio-Chem to NT Theory

- “Slip bond” is a bond that shortens its lifetime in response to tensile force.^108^
  - It is represented by a direct NT from the minimum to SP_1_ (without or sometimes with a TP).
- “Catch bond” is a bond that prolongs its lifetime in response to tensile force.^108^
  - It is often represented by a divided NT, whose division is conveyed by a VRI point between the minimum well and the dissociation channel, for example. The corresponding separatrix is a singular NT. The direction of the external force does not point along the TS valley. The curvature at SP_1_ across the col direction is often larger than the curvature at the minimum well in the same direction.
- “*f*-switch point”^14^ should mark the beginning of catch bond behavior. We reject its reasoning. We will discuss this in a next paper.
  - One could test the special NT at the border where the lowest TP occurs first. But nevertheless, this is often still a slip direction.
- “Ideal bond”^40^ should depict the step from slip to catch direction. We question its possibility—or at least its probability. There does not exist a known example.
  - “**n̂** -switch point”^14^ is the long known VRI point. The singular NT belonging to the VRI point marks the border to the possible catch bond behavior. For problems with more than 2 dimensions, there are manifolds of VRI points.^16,18,20,97,99^
- “Crossing of an NT with Det(**H**) = 0” — barrier breakdown point (BBP), thus the green lines in the figures above are the BBP curves. For problems with more than 2 dimensions, there are manifolds of BBP points.^25,115^
- “Tug-of-war problem” — two competing TSs with a VRI point in between. An external force can select one reaction direction. Of course, a slip can occur in both reaction valleys as well as catch directions for an excitation with their corresponding NTs.

## 6 Conclusions

Applying the NT theory would probably make it easier to understand the tug-of-war problem. By jumping back and forth between slip and catch bond directions, one could change the corresponding reaction direction, especially when one works with a directed force.^88^

We observe in this paper direct or delayed catch bond behavior where we count for only the barrier height of the effective PES after an external excitation. In our opinion, this indicator is the most important of the catch bond behavior. Note that we use for the models simple 2-dimensional toy surfaces. They are good enough to represent some interesting observations for catch bonds in the experiments of the last 35 years.

However, one should note that the model of eq 1 is only a linear approach for the force; therefore, it is the simplest possible model. In refs (24,25), we start to treat a nonlinear approach to an external force. And one should also note that the rules for catch bond behavior, given here, are of empirical character. According to our assumption, it is not enough to look on a PES. No, one needs a control calculation along the NT of the direction of the external force. We are sure that the proposed *f*-switch points of Barkan and Bruinsma^5^ are not the solution.

Of course, the definition of the difference is very important between slip and catch regions for the influence of an external force. However, note that another central intention for the slip force is to find an optimal direction to achieve a BBP with the least amount of force. This is discussed in the articles.^25,115,116^

## Data Availability

All data and Mathematica files are available by e-mail from WQ.

## Appendix

### PES Formulas and Tables

(A) We treat the PES of E-selectin.^5^ We use the constants *k*_θ_ = 273 [pN nm], *D*_0_ = 207 [pN nm], *d*_0_ = 0.26 [nm], σ = 0.18π [radians], θ_0_ = 0.58π [radians], θ_1_ = π [radians], *c* = 9.5 [pN nm], and we use an angle θ and set

with

and build the PES

6

The last summand means force direction (1, 1) for the dashed NT. The normalization by  is suppressed for simplicity; it should be observed for the amount *F*. *F* (1, 1) means the same like . The direction is changed for other NTs (Table 1).

**Table 1:** Stationary Points to PES *V*_eff_ of *V* for E-Selectin by Eq 6, to the NT to Direction , Energies in pN

*F*	Min (*L*, *d*) [nm]	*V*_Min_ [pN]	SP (*L*, *d*) [nm]	*V*_SP_ [pN]	barrier	Δ*x̃*
0	(5.417, 0)	–57.893	(4.425, 9.925)	0	57.893	0
10	(5.430, 0.002)	–112.137	(4.566, 0.791)	–55.256	56.881	–0.372
20	(5.441, 0.004)	–166.525	(4.692, 0.656)	–108.686	57.839	1.955
30	(5.450, 0.007)	–221.037	(4.801, 0.695)	–162.381	58.656	1.846
40	(5.458, 0.009)	–275.658	(4.894, 0.561)	–216.628	59.030	0.512
50	(5.466, 0.011)	–330.379	(4.973, 0.539)	–271.464	58.915	–1.765
60	(5.472, 0.013)	–385.190	(5.040, 0.524)	–326.849	58.341	–4.709
70	(5.478, 0.015)	–440.084	(5.097, 0.512)	–382.719	57.365	

PES of L-selectin^5^

We use the constants *k*_θ_ = 266, *D*_0_ = 237, *d*_0_ = 0.29, σ = 0.12π, θ_0_ = 0.58π, θ_1_ = π, *c* = 0 and use the same formula like above for E-selectin

7

Stationary points on the surface under external excitation in direction  (1, 1) are given in Table 2, together with the corresponding energies. (Compare Figure 2A of ref (5) which we can confirm.).

**Table 2:** Stationary Points to PES *V*_eff_ of *V* for L-Selectin by Eq 7

*F*	Min (*L*, *d*) [nm]	*V*_Min_ [pN]	SP (*L*, *d*) [nm]	*V*_SP_ [pN]	barrier
0	(5.570, 0)	–39.24	(5.151, 0)	10.96	50.194
10	(5.571, 0.002)	–94.95	(5.113, 0.024)	–40.47	54.480
20	(5.571, 0.004)	–150.69	(5.078, 0.075)	–91.87	58.823
30	(5.572, 0.006)	–206.47	(5.093, 0.161)	–143.84	62.629
40	(5.573, 0.009)	–262.26	(5.147, 0.242)	–197.06	65.207
50	(5.573, 0.011)	–318.09	(5.205, 0.302)	–251.55	66.535
60	(5.574, 0.013)	–373.94	(5.225, 0.345)	–307.11	66.836
70	(5.574, 0.015)	–429.82	(5.298, 0.377)	–363.50	66.324
80	(5.575, 0.018)	–485.73	(5.334, 0.400)	–420.55	65.178
90	(5.575, 0.020)	–541.67	(5.364, 0.416)	–478.13	63.537
100	(5.575, 0.023)	–597.63	(5.390, 0.426)	–536.12	61.513

(B) The PES for the linear one-channel approach for a catch bond is

8

It is an abstract model: its scaling is free, as well as the scaling of the next examples. The minimum of this PES is at (0, 0) and its SP at (2, 0). The eigenvalues are (6, 2) and (−6, 10) thus the curvature across the reaction valley is stronger at the TS than at the reactant. The stationary points on the surface under external excitation in *y* direction are given in Table 3, together with the corresponding energies.

**Table 3:** Stationary Points to PES *V*_eff_ of *V* by Eq 8

*F*	Min (*x*, *y*)	*V*_Min_	SP (*x*, *y*)	*V*_SP_	barrier
0	(0, 0)	0.0	(2, 0)	4.0	4.0
1	(0, 0.5)	–0.25	(2.01, 0.1)	3.83	4.08
2	(0, 1.0)	–1.0	(2.03, 0.2)	3.8	4.8
3	(0, 1.5)	–2.25	(2.05, 0.29)	3.56	5.81
4	(0, 2.0)	–4.0	(2.09, 0.37)	3.23	7.23

(C) The PES for a curvilinear one-channel approach for a catch bond is

9

It is used along a proposal of ref (4). The stationary points of the NT along the direction (0.55, 0.83) on this surface are given in Table 4, together with the corresponding energies.

**Table 4:** Stationary Points to PES *V*_eff_ of *V* by Eq 9

*F*	Min (*x*, *Q*)	*V*_Min_	SP (*x*, *Q*)	*V*_SP_	barrier
0	(2.01, 2.25)	–60.0	(2.81, −1.75)	60.0	120.0
10	(4.76, 311)	–133.2	(3.15, −1.54)	54.91	188.1
20	(8.43, 4.02)	–251.7	(3.63, −1.38)	42.94	294.6
30	(13.08, 4.96)	–425.6	(4.18, −1.26)	23.72	449.4
40	(18.76, 5.92)	–665.5	(4.77, −1.15)	–2.93	662.6
50	(25.47, 6.90)	–981.9	(5.39, −1.06)	–37.09	944.8

(D) A case of an emerging new minimum for the PES of Figure 13 under force: The example is again a theoretical model. For the quite skew 2D PES with **x** = (*x*, *y*), we use a product of two quadratic forms similar to refs (6 and 23).

10

The last expression in the definition is used to tighten the col through the TS, because we need it for a putative catch bond. Table 5 reports the stationary points of the PES.

**Table 5:** Stationary Points to PES *V*_eff_ of *V* by Eq 10

*F*	Min (*x*, *y*)	Min2 (*x*, *y*)	SP (*x*, *y*)	barrier
0	(−1.00, −1)		(0.59, 0.04)	27.92
1	(−0.99, −1)		(0.59, 0.04)	26.34
2	(−0.97, −1)		(0.59, 0.04)	24.77
3	(−0.96, −1)		(0.59, 0.03)	23.21
4	(−0.95, −1)		(0.59, 0.03)	21.66
5	(−0.93, −1)	(0.49, −0.81)	(0.60, 0.03)	20.13
6	(−0.92, −1)	(0.58, −0.73)	(0.60, 0.03)	18.61
7	(−0.90, −1)	(0.63, −0.69)	(0.60, 0.03)	17.10
8	(−0.88, −1)	(0.66, −0.69)	(0.61, 0.02)	15.60
9	(−0.86, −1)	(0.69, −0.66)	(0.61, 0.02)	14.12
10	(−0.84, −1)	(0.71, −0.64)	(0.61, 0.02)	12.66
11	(−0.82, −1)	(0.72, −0.64)	(0.61, 0.02)	11.21
12	(−0.80, −1)	(0.74, −0.63)	(0.62, 0.02)	9.89
13	(−0.77, −1)	(0.76, −0.63)	(0.62, 0.01)	9.81
14	(−0.75, −1)	(0.77, −0.63)	(0.62, 0.01)	9.81
15	(−0.71, −1)	(0.78, −0.62)	(0.63, 0.01)	9.86
16	(−0.67, −1)	(0.79, −0.62)	(0.63, 0.01)	9.93
17	(−0.62, −1)	(0.80, −0.62)	(0.63, 0.01)	10.02
18		(0.82, −0.62)	(0.64, 0.01)	10.12
19		(0.83, −0.61)	(0.64, 0.0)	10.23
20		(0.84, −0.61)	(0.64, 0.0)	10.36
30		(0.93, −0.61)	(0.68, −0.01)	13.81
50		(1.08, −0.62)	(0.74, −0.02)	19.79

(E) The example for a tug-of-war mechanism

The theoretical formula of the PES of example (B) is changed by a Morse ascent to the positive *y*-direction, thus we assume in *y*-direction a bond length with a Morse behavior. The former one-channel approach is turned into a two-channel one because now at (0, ∞) emerges a second TS.

11

The former minimum is again at (0, 0) and its SP at (2, 0). The eigenvalues are (6, 20) and (−6, 27), thus the curvature across the reaction valley is still stronger at the TS than at the reactant. The stationary points on the surface under external excitation in direction (0.24, 0.97) along the fat dashed NT are given in Table 6, together with the corresponding energies. Note that the left upper part of the table is similar to Table 3.

**Table 6:** Stationary Points to PES *V*_eff_ of *V* by Eq 11

*F*	Min (*x*, *y*)	SP (*x*, *y*)	Barr	P2 (*x*, *y*)	Barr2
0	(0, 0)	(2.0, 0)	4.0	(0, ∞)	10.0
1	(0, 0.05)	(2.0, 0.038)	4.001	(0, 2.94)	6.03
2	(0, 0.11)	(2.01, 0.082)	4.033	(0, 2.18)	3.62
3	(0, 0.20)	(2.02, 0.132)	4.086	(0, 1.69)	1.85
4	(0, 0.32)	(2.04, 0.190)	4.184	(0, 1.29)	0.62

(F) The PES with four minima for a tug-of-war mechanism

We use a modified Wolfe-Quapp surface^117^

12

The modification concerns the summand with the exponential term in the second line. At the TS1, an additional strong hill is included which enforces a second maximum of the PES model. For a 2D PES, the maxima are saddles of index two which are common in theoretical chemistry.^118,119^ Stationary points, energies, and barriers are given in Table 7.

**Table 7:** Stationary Points to PES *V*_eff_ of Eq 12^a^

*F*	Min (*x*, *y*)	SP_1_ (*x*, *y*)	barrier
0	(1.23, −1.57)	(−0.25, −1.50)	3.169
1	(1.22, −1.52)	(−0.25, −1.49)	3.120
2	(1.22, −1.47)	(−0.25, −1.49)	3.117
3	(1.22, −1.41)	(−0.25, −1.48)	3.166
4	(1.21, −1.34)	(−0.25, −1.48)	3.274
5	(1.20, −1.26)	(−0.25, −1.47)	3.452
6	(1.19, −1.15)	(−0.25, −1.47)	3.718

a With *F* = 3, the barrier increases.

## Abbreviations

NT: Newton trajectory
PES: potential energy surface
SP, TS: saddle point, transition state
TP: turning point
VRI: valley-ridge inflection point
